# Temporal binding of auditory spatial information across dynamic binaural events

**DOI:** 10.3758/s13414-017-1436-0

**Published:** 2017-10-30

**Authors:** G. Christopher Stecker

**Affiliations:** Department of Hearing and Speech Sciences, Vanderbilt University School of Medicine, 1215 21st Avenue South, Rm 8310, Nashville, TN 37232 USA

**Keywords:** Spatial localization, Audition, Multisensory processing

## Abstract

Simultaneity judgments were used to measure temporal binding windows (TBW) for brief binaural events (changes in interaural time and/or level differences [ITD and ILD]) and test the hypothesis that ITD and ILD contribute to perception via separate sensory dimensions subject to binding via slow (100+ ms)—presumably cortical—mechanisms as in multisensory TBW. Stimuli were continuous low-frequency noises that included two brief shifts of either type (ITD or ILD), both of which are heard as lateral position changes. TBW for judgments within a single cue dimension were narrower for ITD (mean = 444 ms) than ILD (807 ms). TBW for judgments across cue dimensions (i.e., one ITD shift and one ILD shift) were similar to within-cue ILD (778 ms). The results contradict the original hypothesis, in that cross-cue comparisons were no slower than within-cue ILD comparisons. Rather, the wide TBW values—consistent with previous estimates of multisensory TBW—suggest slow integrative processing for both types of judgments. Narrower TBW for ITD than ILD judgments suggests important cue-specific differences in the neural mechanisms or the perceptual correlates of integration across binaural-cue dimensions.

**Electronic supplementary material:**

The online version of this article (10.3758/s13414-017-1436-0) contains supplementary material, which is available to authorized users.

The *temporal binding window* (TBW) is a powerful construct for studying how brain mechanisms combine information across sensory dimensions (Wallace & Stevenson, 2014). It reflects the propensity for simultaneous/integrated perception when multiple sensory events (e.g., auditory tone and visual flash) occur within a critical temporal interval (the TBW). Importantly, the TBW is not universal but varies with the sensory dimension(s) involved, and whether events occur on the same or different (i.e., independent) dimensions. This study measured TBW across two dimensions of binaural auditory information to understand how those dimensions are integrated to form representations of auditory space.

The perceptual representation of auditory space is poorly understood, in part because—unlike in touch or vision—space is not directly represented on the sensory epithelium. Rather, it must be computed by integrating multiple informative but imperfect cues. Spatial acuity is highest in the horizontal dimension, where interaural time and level differences (ITD and ILD; see Stecker & Gallun, 2012) are the dominant cues. ITD and ILD are initially extracted by parallel brainstem mechanisms, but there is significant debate as to whether the cues combine “early” to form a single sensory dimension of auditory space in the early auditory pathway, or instead maintain quasi-independent representations that combine “late”—for example, via cortical mechanisms. If the latter, do ITD and ILD comprise discrete sensory dimensions subject to binding via the same principles (e.g., the TBW) that govern multisensory integration? The current study exploited differences in TBW for judgments within versus across dimensions to address this question. Specifically, we adapted the simultaneity-judgment paradigm (Dixon & Spitz, 1980) to measure TBW for ITD-based and ILD-based events.

The study paradigm is illustrated in Fig. 1. When observers judge the perceived simultaneity of events separated by a variable stimulus onset asynchrony (SOA), a peaked function of SOA is revealed (Fig. 1a–b). The width of that function estimates the TBW, that is, the tolerance of temporal mismatch, and hence the temporal acuity of the simultaneity judgment. Multisensory TBW estimates, which range from 200 to 600 ms and are thought to reflect cortical processing, vary significantly across individuals (Stevenson, Zemtsov, & Wallace, 2012), during development (Hillock, Powers, & Wallace, 2011), and with training (Powers, Hillock, & Wallace, 2009). Importantly, TBW estimates also vary as a function of the sensory dimensions involved, reflecting the underlying temporal acuity of each but limited mainly by the poorer of the two (cf. Burr, Banks, & Morrone, 2009). Simultaneity judgments also reveal greater temporal acuity (i.e., narrower TBW) when events occur on the same versus different sensory dimensions. For example, Bartels and Zeki (2006) found wider TBW for visual Color+Motion changes (544 ms) than for two color changes (328 ms) or two motion changes (269 ms). Similarly, Fujisaki and Nishida (2010) reported synchronous perception of unidimensional visual events up to 7–9 Hz (100–150-ms SOA) but only 3–5 Hz (200–300-ms SOA) for auditory-visual multisensory events.

**Fig. 1:**
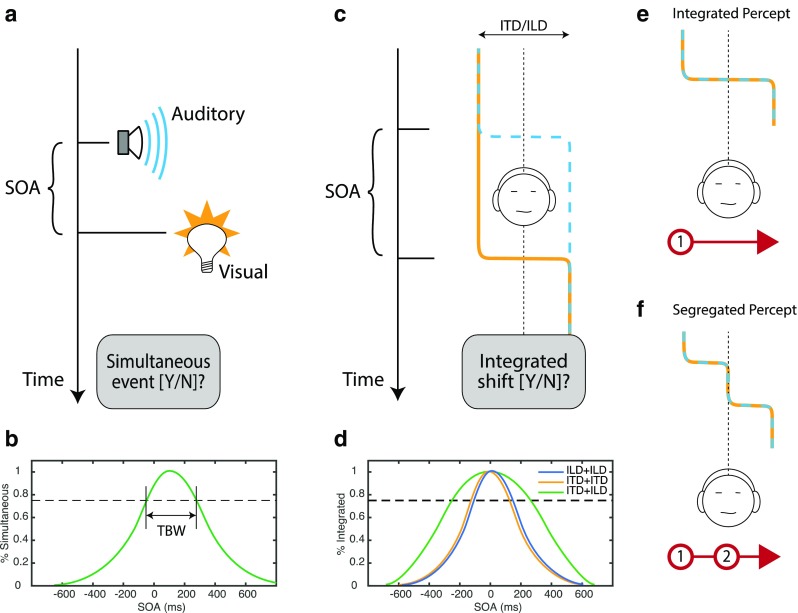
Simultaneity-judgment paradigm for multisensory event perception (**a**) asks observers to indicate whether two events separated by a brief interval (SOA) are perceived as occurring simultaneously. TBW is estimated by plotting response likelihood against SOA and taking the width of this function (**b**). Current paradigm (**c**) modifies this approach by presenting two binaural-change events. For example, the ITD (yellow line) and ILD (blue dashed line) initially favor the left ear. After a variable delay, the ILD shifts to favor the right ear, followed (after the SOA) by the ITD. The resulting percept can take one of two forms–integrated or segregated–and broader TBW (**d**) was hypothesized for between-cue (green) than within-cue (yellow, blue) judgments. At short SOA, the integrated percept (**e**) tends to dominate. The image moves from left to right in a single step. Listeners indicate the percept by drawing one line (red) on a touch display. At longer SOA, the segregated percept (**f**) becomes likely. The image moves in two steps, pausing briefly near the middle of the head. Listeners draw two lines, one per shift in perceived location

In this study, we replaced the auditory and visual events of Fig. 1a with brief changes (perceived as lateral “shifts”) in ILD and/or ITD on each trial (Fig. 1c). Note that unlike in past studies that measured TBW across stimuli in separate low-level channels (e.g., different visual locations), the current study measured TBW across sensory features (ITD and ILD) applied to a single stimulus (common frequency range). We hypothesized that within-cue trials (two ILD shifts or two ITD shifts) would reveal narrow TBW similar to other unisensory estimates (Fig. 1d). Across-cue trials (presenting one ITD and one ILD shift) would provide a critical test: If integration occurs early so that ITD and ILD impact a single perceptual dimension, TBW should remain narrow. Significant broadening of the TBW for cross-cue trials would suggest late integration; depending on the TBW range (i.e., if similar to multisensory TBW), slow—presumably, cortical—processes could be implicated in binaural-cue integration.

## Method

### Participants

Twelve (eight female) adult listeners (ages 22–44 years) with normal hearing (audiometric thresholds <20 dB HL in each ear at all octave frequencies 250–8000 Hz, differing by <10 dB across ears at any frequency) participated. One was the author and three others were research assistants affiliated with the lab but not informed about the study purpose or hypotheses. Others were paid participants naive to the purpose of the study. Two outliers (see Results section) were excluded from final analyses, which thus involved 10 participants.

### Stimuli

One-octave flat-spectrum bands of noise centered at 500 Hz were synthesized at 48 kHz sampling rate in MATLAB (R2014b, MathWorks, Natick MA) were presented over electrostatic headphones (SR-307, Stax Ltd, Saitama Japan) at 70 dB SPL (A-weighted).

The total duration of each stimulus was 2,000 ms, including 20-ms cos^2^ on/off ramps. Two “shifts” in ITD and/or ILD were applied partway through this duration, with overall timing randomized to reduce predictability. To avoid audible artifacts, shifts were implemented as smooth changes over 10 ms (1/4-cycle cosine profile). One shift, which we label Shift A, occurred at a random time between 800 and 1,200 ms following sound onset. The other (Shift B) preceded or followed Shift A by an SOA of −600, −400 −200, −100, −50, 0, 50, 100, 200, 400, or 600 ms. Positive values indicate that Shift A preceded Shift B and negative values indicate the opposite order.

Shift values are indicated in Table 1. Three conditions were tested. In condition ILD+ILD, each shift changed the ILD by 6 dB. In condition ITD+ITD, each shift changed the ITD by 500 μs. In condition ITD+ILD, Shift A changed the ITD by 500 μs and Shift B changed the ILD by 6 dB. In all cases, the two shifts were applied in the same direction (left to right or right to left). Shifts were always balanced across the midline, so the starting configuration favored one of the ears by A/2 + B/2 and the ending configuration favored the opposite ear by the same amount. Thus, in condition ILD+ILD, the ILD initially favored the left ear (for example) by 6 dB. After the first shift, the ILD was 0 dB, and following the second shift, ILD favored the right ear by 6 dB, a total excursion of 12 dB. In condition ITD+ILD, the stimulus initially favored the left ear by 3 dB + 250 μs, and ended favoring the right ear by the same amount. During the SOA, the cues favored opposite ears, by 3 dB ILD to one ear and 250 μs ITD to the other.

**Table 1:** Stimulus values in each condition

	Condition		
	ILD + ILD	ITD + ITD	ITD + ILD
Shift A	6 dB	500 μs	500 μs
Shift B	6 dB	500 μs	6 dB
Total	12 dB	1000 μs	500 μs + 6 dB

*Note.* Values indicate the unsigned magnitude of each shift in ITD or ILD. Bottom row lists the total shift from beginning to end of each trial

The stimulus values used in this study (±3 dB and ±250 μs) correspond roughly to acoustical measurements at 500 Hz for sources near 20° azimuth (Diedesch, 2016; Feddersen, Sandel, Teas, & Jeffress, 1957; Kuhn, 1977). The values were selected to produce suprathreshold lateralization that would not saturate at (A/2 + B/2) in any condition. That is, the starting/ending values and the shifts themselves were set to be clearly detectible in all conditions. Initial pilot testing confirmed that listeners could discriminate the direction of each such stimulus at close to 100% correct. Pilot testing also confirmed that ILD and ITD values produced roughly similar lateral percepts.

### Task procedures

Task procedures were designed to measure perceived simultaneity along with perceived lateral position. For this purpose, participants indicated stimulus trajectories on a touch-screen interface (iPad Air, Apple Inc., Cupertino CA) that displayed a reference schematic head and center line. On each trial, participants “drew” one or two arrows on the display as indicated in Figs. 1e–f. Integrated percepts were indicated by a single arrow indicating the starting and ending lateralization. Segregated percepts were indicated by two arrows marking the start and end of each shift.

Participants were introduced to the task sequentially by training on (1) static localization, (2) indication of single shifts, (3) indication of two easily segregated shifts (at long SOA), and (4) identification of integrated/segregated percepts (one or two arrows). Once listeners expressed comfort with the full task, experimental testing commenced with the full range of conditions and SOA values. Each participant then completed four runs of 44 trials each (a total of eight repetitions per SOA value) in each condition.

**Fig. 2:**
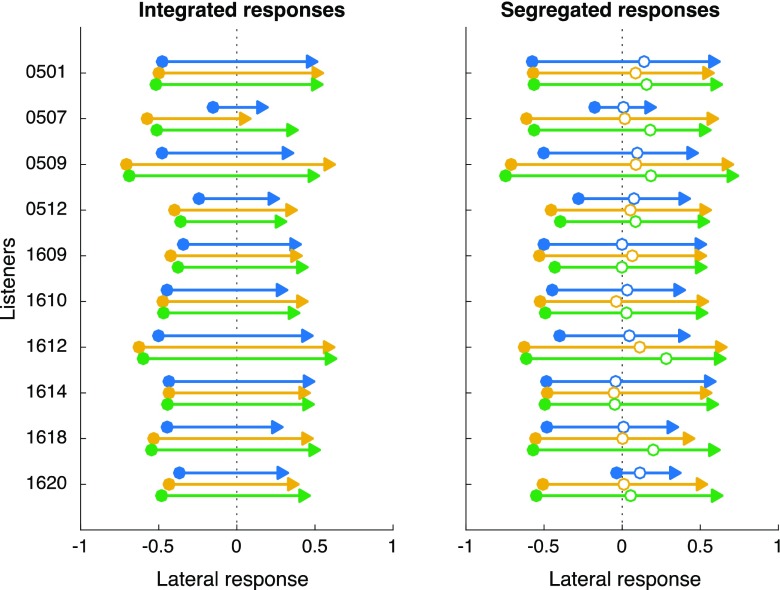
Lateralization data. Each arrow indicates the mean position of touch responses corresponding to sound onset (filled circles) and sound offset (triangles). For nonintegrated responses, open circles indicate judgments of the end of the first shift/beginning of the second (i.e., the position at which listeners heard the image “rest” before continuing). Lateral positions are given in screen units ranging from −1 (left edge) to +1 (right edge). Data are shown progressing from left to right; right-to-left trials have been flipped prior to averaging. For each listener (groups of three lines), data are plotted separately for ILD+ILD (top blue line), ITD+ITD (middle yellow line), and ITD+ILD (bottom green line) conditions. Panels separately plot response locations on trials that listeners labeled as integrated (left) or not (right)

**Fig. 3:**
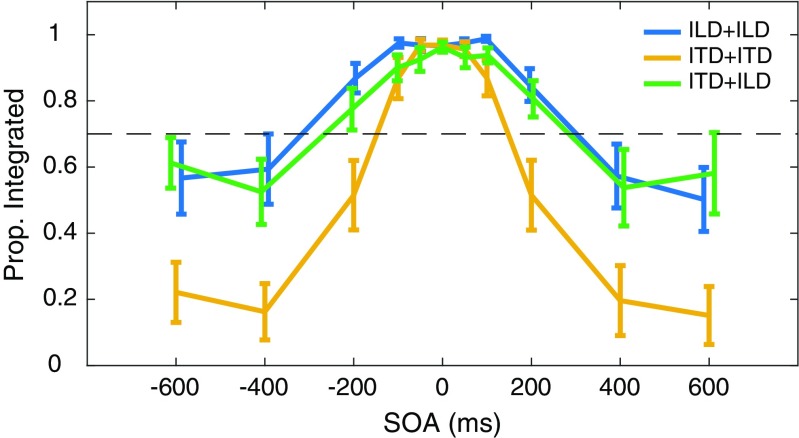
Proportions (*y*-axis) of trials indicated as “integrated” are plotted as a function of SOA (*x*-axis) in each condition (lines). Points plot the mean across listeners for each trial type. Error bars indicate the corresponding standard error. Dashed line indicates proportion (0.7) used to quantify TBW in Fig. 4

**Fig. 4:**
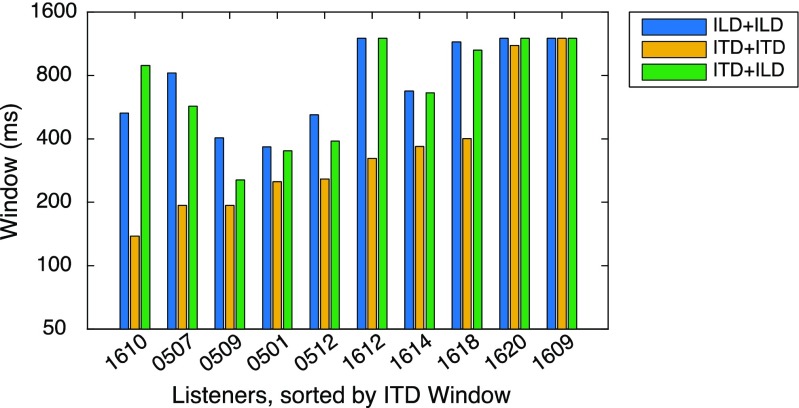
TBW by listener and condition. Data are sorted by TBW in the ITD+ITD condition. Across a wide range of overall TBW, most listeners exhibited narrower TBW in the ITD+ITD condition than in either of the other conditions (both of which included ILD shifts)

Analysis of response data began with confirmation of accurate identification of starting position and shift direction (initial touch left or right of midline; see Fig. 2). Second, listeners’ report of integrated versus segregated perception (i.e., whether they drew one or two arrows in response on each trial) was used to compute the TBW for each participant and condition. The mean proportion of trials reported as integrated (response = one arrow) was computed as a function of SOA. A Gaussian was then fit to each such response function with center (constrained to ±200 ms), height, and width (both unconstrained) as free parameters. TBW was calculated as the span exceeding 70% “integrated” responses. Grand mean response functions, along with standard error across participants, are plotted in Fig. 3. Values of TBW from Gaussian fits are plotted across participants and conditions in Fig. 4. Individual response data with Gaussian fits are plotted in Supplemental Fig. S1. Single-factor repeated-measures ANOVA was used to evaluate group-wise differences in TBW across conditions.

## Results

Two listeners were identified as outliers and eliminated from further analysis. One indicated “integrated” perception on fewer than 50% of trials with 0-ms SOA (i.e., physically simultaneous shifts). The other was unable to achieve 85% correct identification of the side (left or right) to which stimuli initially appeared and thus could not reliably judge the direction of shifts. In both cases, data exceeded the mean of other participants by more than 3σ.

Correct lateralization was confirmed by comparing the sign (left or right) of initial touch response to the starting ITD or ILD. Lateralization of single shifts was confirmed at 100% correct during initial training, except for listener 1614, who made a left/right error on one of 12 ILD trials. Initial lateralization of two-shift stimuli in the main experiment was similarly accurate: mean lateralization was 99% correct (σ = 1.6%) and did not significantly differ between conditions, *F*(2, 18) = 2.68, *p* = .1, confirming that ITD and ILD values were presented at clearly identifiable levels. Figure 2 illustrates that the left/right position of touch responses differed slightly but significantly across conditions. Initial touch position (filled circles) fell closer to midline in the ILD+ILD condition (mean = 0.40 of maximum) than in the ITD+ITD (mean = 0.53), *F*(1, 9) = 10.47, *p* < .05, or ITD+ILD (mean = 0.51), *F*(1, 9) =1 0.05, *p* < .05. The result indicates slightly (~20%) weaker lateralization of the 6 dB starting ILD than the 500 μs starting ITD. This issue is taken up in the Discussion section.

Figure 3 plots the proportion of “integrated” responses, as a function of SOA and averaged across listeners, in each condition. The result is immediately evident and inconsistent with the hypothesis that across-cue judgments would produce wider TBW. Rather, data from across-cue judgments (ITD+ILD) appear virtually identical to those from within-cue ILD+ILD judgments. Both conditions elicited higher proportions of “integrated” responses at larger SOA values than did within-cue ITD+ITD judgments, consistent with narrower TBW for ITD than for ILD judgments.

The TBW difference between ITD+ITD and other conditions, along with the lack of difference between ITD+ILD and ILD+ILD data, is further illustrated by plotting TBW across participants and conditions in Fig. 4 (also see Supplemental Fig. S1). Despite significant individual variation in overall TBW, the effect of condition was quite robust: TBW was shorter in ITD+ITD (mean = 444 ms) than in ILD+ILD (mean = 807 ms), *F*(1, 9) = 15.01, *p* < .05, or ITD + ILD (mean = 778 ms), *F*(1, 9) = 10.95, *p* < .05, conditions. Two participants (1609 and 1620) exhibited TBW durations >1000 ms in all conditions. Despite good identification of lateral position and shift direction (left or right), these listeners indicated “integrated” perception on the vast majority of trials. Excluding them from the analysis reduced mean TBW (to 709, 266, and 672 ms in ILD+ILD, ITD+ITD, and ITD+ILD, respectively) but did not alter any statistical conclusions.

## Discussion

### Binaural TBW values greatly exceed other estimates of auditory temporal resolution

The overall range of TBW (150–800+ ms) was roughly consistent with multisensory TBW (e.g., Stevenson et al., 2012) and well in excess of estimates from auditory temporal tasks such as gap detection (10–50 ms: Lister, Besing, & Koehnke, 2002; Phillips, Hall, Harrington, & Taylor, 1998; Phillips, Taylor, Hall, Carr, & Mossop, 1997; Pollack, 1977), temporal order judgments (10–30 ms: Divenyi & Hirsh, 1974), and duration discrimination (Abel, 1972; Creelman, 1962). The difference, which suggests poorer temporal resolution for dynamic binaural events than for other types of auditory events, is consistent with “binaural sluggishness” noted in past studies. Blauert (1972) and Grantham and Wightman (1978), for example, found that listeners could not follow periodic ITD modulations faster than 2–5 Hz. Grantham (1984) reported similar data for ILD modulations, although two listeners in that study were able to follow rates up to 20 Hz, suggesting that in some cases ILD processing could be more temporally efficient. That difference is opposite to the current results, but the overall range of TBW is consistent with Grantham’s (1986) suggestion that it takes at least 150–300 ms for the auditory system to determine the lateral position of a dynamic sound.

### Cross-cue TBW did not exceed within-cue TBW, but TBW differed between cue conditions

The results provide no evidence of longer TBW duration in across-cue versus within-cue simultaneity judgments. Rather, the consistently long TBW for ITD+ILD and ILD+ILD suggests that slow integrative mechanisms are involved in both types of judgments. The close match between those two conditions further suggests that cross-cue judgments were limited primarily by the ILD shifts as the sensory dimension with poorer temporal acuity (cf. Burr et al., 2009). Two potential explanations may be considered for the difference between ITD+ITD and ILD+ILD:

First, notwithstanding Grantham’s (1984) opposite result, the temporal processing of ILD changes could be intrinsically slower than for ITD changes. For example, ILD might require longer temporal integration to smooth out fluctuations in sound level. Indeed, brain-stem physiology suggests slower ILD integration versus greater transient sensitivity to ITD change (Remme et al., 2014). On their own, those differences are too small to account for the differences found here, but may become exaggerated along the auditory pathway (Brown & Tollin, 2016). In fact, whereas ITD can only be computed via early brainstem mechanisms with microsecond acuity, ILD cues could be computed de novo via binaural comparisons in slower midbrain and cortical structures that encode sound level via spike rates. Such mechanisms, which would expand the relative TBW for ILD processing, have been invoked previously to account for recency effects in ILD-based but not ITD-based lateralization (Stecker & Hafter, 2009; Stecker, Ostreicher, & Brown, 2013).

The possibility of cue-specific differences should be mediated by consideration of the low-frequency range (~500 Hz) employed here. ITD sensitivity is particularly potent at 500 Hz compared to frequencies >1500 Hz, whereas ILD sensitivity is roughly invariant with frequency from 250 to 10000 Hz (Mills, 1960). For this and other reasons, ITD is primarily dominant at low frequencies, and temporal differences between ITD and ILD could plausibly vary across frequencies with different patterns of cue dominance.

Second, the two types of shifts could have induced different spatial percepts (e.g., in extent of lateralization). Although acoustical measures (Diedesch, 2016; Kuhn, 1977) and pilot testing both suggested similar lateral extent of the selected ITD and ILD values, the lateralization data (see Fig. 2) suggest that several participants perceived images closer to the midline in condition ILD+ILD than in ITD+ITD. Bearing in mind that psychophysical sensitivity to the cues was very high (response lateralization was 99% correct and did not vary across conditions) and unlikely to be masked at SOA values of 200–600 ms, could the *perceived* location of ILD shifts have directly influenced their perceived timing? That is, are larger temporal separations necessary to compensate for smaller spatial separations and vice versa? Although the relevant literature remains incomplete, some recent studies suggest that stimulus magnitude modulates temporal processing in just this way. In particular, Krueger Fister et al., (2016) demonstrated greater perception of audiovisual synchrony for low-intensity versus high-intensity auditory (signal/noise: 5 vs. 23 dB) and visual (luminance contrast: 24.4 vs. 766.8) stimulus pairs. Unlike in the current study, those stimulus differences were large enough to significantly impact unisensory performance. The difference in TBW (294 vs. 260 ms, based on Gaussian fit to the published data) was much smaller than found in the current study, but consistent (in direction) with temporally broader integration of less potent stimuli. It could be the case that auditory spatial perception drives such differences more strongly than do differences in simple intensity, but additional work will be necessary to identify and understand such contributions.

## Summary and conclusions

This article is the first to measure temporal binding windows for auditory spatial features distributed on two key dimensions of binaural information: ITD and ILD. The simultaneity judgment paradigm, adapted from multisensory experiments, provided estimates of TBW for integration within and across two types of events: ITD changes and ILD changes. The following conclusions are supported by the results:

1. Estimates of TBW within and across binaural dimensions ranged from 150 to 1200 ms, broadly consistent with TBW estimates for perceived simultaneity in multisensory event pairs. The TBW estimates are consistent with the temporal profile of “binaural sluggishness” (Grantham, 1984) but far exceed estimates of temporal processing in monaural auditory tasks.
2. Narrower TBW estimates were obtained for judgments of ITD-change events compared to ILD-change events. Future studies should resolve whether that difference reflects (a) intrinsic differences between ITD and ILD processing due to the nature of the physical cue or its perceptual weighting in different frequency regions, or (b) trade-offs between perceived temporal and spatial separation in high-level auditory scene analysis.
3. TBW estimates were nearly identical for simultaneity judgments within the ILD dimension or across dimensions (ITD+ILD), consistent with dominance of the slower dimension (ILD). The result provides no evidence for slower integration across versus within binaural-cue dimensions.

## Electronic supplementary material

Fig. S1 Data for individual listeners. Each panel plots the proportion of trials indicated as “integrated” (*y*-axis) as a function of SOA (*x*-axis). Response data (symbols), along with Gaussian fits (curves) are plotted for condition ILD+ILD (blue), ITD+ITD (yellow), and ITD+ILD (green). Dashed line indicates proportion (0.7) used to quantify TBW in Fig. 4. Each panel plots data for a different listener (DOCX 432 kb)
