# Supplementary material for: Temporal binding of auditory spatial information across dynamic binaural events
Source: Atten Percept Psychophys. 2017 Oct 30;80(1):14–20. doi: 10.3758/s13414-017-1436-0 (PMC5735209; doi:10.3758/s13414-017-1436-0)
Supplement: Supplementary file 1 — Data for individual listeners. Each panel plots the proportion of trials indicated as “integrated” (y-axis) as a function of SOA (x-axis). Response data (symbols), along with Gaussian fits (curves) are plotted for condition ILD+ILD (blue), ITD+ITD (yellow), and ITD+ILD (green). Dashed line indicates proportion (0.7) used to quantify TBW in Fig. 4. Each panel plots data for a different listener (DOCX 432 kb) [file 13414_2017_1436_MOESM1_ESM.docx]

**Fig. S1** Data for individual listeners. Each panel plots the proportion of trials indicated as “integrated” (*y*-axis) as a function of SOA (*x*-axis). Response data (symbols), along with Gaussian fits (curves) are plotted for condition ILD+ILD (blue), ITD+ITD (yellow), and ITD+ILD (green). Dashed line indicates proportion (0.7) used to quantify TBW in Fig. 4. Each panel plots data for a different listener

**Supplemental Material**
